# Five-Year German PrEP cohort reveals high HIV protection and persistent STI burden: implications for individualized and flexible prevention strategies

**DOI:** 10.1007/s15010-025-02667-w

**Published:** 2025-10-25

**Authors:** Maher Almahfoud, Lukas Weimann, Guido Schäfer, Till Koch, Hanna Matthews, Hanna-Marie Weichel, Friederike Hunstig, Marc Grenz, Robin L. Scheiter, Marylyn M. Addo, Julian Schulze zur Wiesch, Olaf Degen

**Affiliations:** 1 Institute for Infection Research and Vaccine Development (IIRVD), Center for Internal Medicine, University Medical Center Hamburg-Eppendorf, Hamburg, Germany; 2 1st Department of Medicine, Division of Infectious Diseases, Center for Internal Medicine, University Medical Center Hamburg-Eppendorf, Hamburg, Germany; 3 German Center for Infection Research (DZIF), Partner Site Hamburg-Lübeck-Borstel-Riems, Hamburg, Germany; 4 Infektionsmedizinisches Centrum Hamburg, Hamburg, Germany; 5 Department of Pulmonology, Infectious Diseases and Oncology, Klinikum Itzehoe, Itzehoe, Germany; 6 Department of Intensive Care Medicine, University Medical Center Hamburg-Eppendorf, Hamburg, Germany; 7 Hein & Fiete, Prävention E.V., Hamburg, Germany; 8 Albertinen Center for Radiology, Evangelical Amalie Sieveking Hospital, Hamburg, Germany

**Keywords:** Pre-exposure prophylaxis, HIV prevention, Sexually transmitted infections, Real-world cohort study, Substance use, Chemsex

## Abstract

**Purpose:**

To evaluate adherence patterns, effectiveness, and sexually transmitted infection (STI) incidence among pre-exposure prophylaxis (PrEP) users in Germany and identify strategies to optimize HIV and STI prevention through individualized care and alternative PrEP modalities.

**Methods:**

A single-site, pseudonymized prospective cohort study was conducted in Hamburg, Germany from December 2019 to September 2024. Clinical and laboratory data were linked with structured behavioral surveys from PrEP users at the University Medical Center Hamburg-Eppendorf.

**Results:**

Of 980 consented individuals, 589 initiated PrEP (median age 32 years, 97.1% male, and 81.8% were born in Germany). The mean follow-up was 102.3 weeks (IQR: 38.6–151.4), totaling 1189.5 person-years. Daily users averaged 315 days of PrEP coverage per year (IQR: 293.0–361.9 days), whereas on-demand users averaged 219 days (IQR: 138.4–311.6 days), highlighting substantial variability in usage patterns. The overall dropout rate was 46.9%. No cases of HIV occurred during active PrEP use. STI incidence remained high 52.4 /100 PY (95% CI: 47.8–57.4, n = 421) for daily PrEP users, 38.9/100 PY (95% CI: 30.1–49.5, n = 79) for event-driven users, predominantly due to *Chlamydia trachomatis* (21.1/100 PY) and *Neisseria gonorrhoeae* (18.8/100 PY). Interest in long-acting PrEP was high (70%), especially among illicit substance users (OR 5.54). Renal function remained stable during follow-up.

**Conclusion:**

PrEP demonstrated high effectiveness despite heterogeneous risk burden and generally stable renal function. This supports flexible, person-centered models with simplified, risk-stratified monitoring and long-acting options. To extend impact beyond MSM, services should add multilingual access and women- and migrant-inclusive outreach.

**Supplementary Information:**

The online version contains supplementary material available at 10.1007/s15010-025-02667-w.

## Introduction

### HIV prevention

Despite significant advancements in HIV prevention, the global targets for reducing new HIV infections remain unmet. In 2022, over 1.3 million new HIV infections occurred worldwide, falling short of the goal to reduce incidence to below 370,000 by 2025. A key element in The World Health Organization's (WHO) Global Health Sector Strategy on HIV for 2022–2030 is maximizing the use of antiretroviral drugs for prevention, aiming to increase the percentage of at-risk individuals using combination prevention methods from 8% in 2022 to 95% by 2025 [1].

Pre-exposure prophylaxis (PrEP) with Tenofovir disoproxil fumarate/Emtricitabine (TDF/FTC) has proven to be a highly effective method for preventing HIV in individuals with increased risk due to sexual behaviors [2]. Endorsed by WHO since 2012 [3] and included in German-Austrian guidelines for HIV-PrEP [4]. TDF/FTC-based PrEP received approval from the European Medicines Agency (EMA) in 2016 [5]. In Germany, the widespread use of PrEP coincided with the availability of affordable generic TDF/FTC in 2017, further bolstered in 2019 when statutory health insurance began covering oral PrEP prescribed by licensed professionals. The number of PrEP users in Germany increased from an estimated 15,600 in June 2020 [6], to approximately 32,000 by the end of 2022 [7]. This rise is significant, particularly among men who have sex with men (MSM), where the estimated need for PrEP ranges from 49,500 to 109,000 [6].

Considering this background, we aim to provide longitudinal, real-world evidence from a large German PrEP cohort by integrating behavioral, clinical, and laboratory data to quantify PrEP uptake, adherence, and effectiveness; estimate sexual behaviors characteristics and STI risk profiles; examine associations with substance use; better understand the impact of COVID-19 pandemic on PrEP use and sexual behavior, and assess renal safety under TDF/FTC.

### Impact of the COVID-19 pandemic

As of 2021, Germany reported approximately 90,800 people living with HIV, with 90% diagnosed and 96% of those receiving antiretroviral therapy. This has contributed to a steady decline in new HIV infections—from about 3,500 cases in 2006 to around 1,800 in 2019. Most infections (68%) occur among MSM [8]. A temporary decline in HIV incidence was observed during the COVID-19 pandemic (2019–2020), likely influenced by reduced sexual activity and healthcare access. However, this remains a hypothesis requiring further analysis [9]. Post-pandemic trends show a slight increase in HIV incidence among heterosexuals and people who inject drugs, underlining the need for broader and more diverse prevention approaches beyond PrEP [9].

### Long-acting PrEP (LA-PrEP)

Following the initial uptake of oral PrEP, new modalities such as LA-PrEP have been developed to address adherence challenges. LA-PrEP agents like cabotegravir and lenacapavir represents an advancement in HIV prevention. Randomized clinical trials of long-acting injectable PrEP were conducted across multiple countries—among cisgender men who have sex with men and transgender women [10] and among cisgender women [11]—and twice-yearly lenacapavir has demonstrated high efficacy in adolescent girls and young women [12] and in men and gender-diverse persons [13]. [10, 12]Addressing adherence and stigma challenges of oral PrEP, injectable PrEP could offer a more appealing alternative, leading to higher overall adoption of PrEP strategies [14]. In the United States, interest in long-acting injectable PrEP has been particularly high, as demonstrated by national surveys among gay and bisexual men [15, 16]. Similar preferences have also been reported among MSM in the Netherlands [17] suggesting a broad acceptance range and indicating a shift towards favoring LA-PrEP among individuals unwilling or unable to take daily oral regimens [15–17].

### Substance use among PrEP users

Given that adherence and risk patterns are influenced by behavioral factors, we examined sexualized drug use within our cohort, focusing on substances commonly linked to “chemsex,” as prior work has associated PrEP use with elevated rates of substance use [18, 19]. This phenomenon is increasingly reported among MSM, particularly in major metropolitan areas across Europe [20].

### Renal function under TDF/FTC

To complement epidemiologic and behavioral outcomes, we examined renal function trajectories under TDF/FTC in real-world care, an area where prior studies have reported mixed findings. While several scientific evaluations have reported a significant decline in renal function associated with long-term use of TDF-based regimens [21, 22], other studies have found no clinically relevant impact on renal function, particularly among younger individuals [23]. This study aims to expand the evidence base on renal safety by contributing longitudinal data from a German PrEP cohort.

To our knowledge, such prospective, pseudonymized real-world data remain scarce, especially in Germany; this study addresses that gap by linking individual-level clinical and laboratory data with repeated behavioral surveys in routine care.

## Methods

### Study population and data collection:

A prospective observational cohort study was conducted at the Outpatient Department of Infectious Diseases of the University Medical Center Hamburg-Eppendorf. The study was initiated in October 2020 and is ongoing. During routine PrEP and STI consultations at our sexual health checkpoint, clinicians and trained peer counselors actively invited all consecutive, eligible patients to participate. Eligibility criteria included being HIV-negative at enrollment, residing in Germany, being at least 18 years old, and proficiency in German. Participation was voluntary; declining had no impact on clinical care.

PrEP indication was assessed by the treating physicians at baseline and at each follow-up in accordance with the German–Austrian PrEP guideline [4], scheduling an initial consultation and quarterly follow-up consultations by experienced physicians for infectious diseases, which included a complete blood count, estimated glomerular filtration rate (eGFR) based on serum creatinine, 4th generation HIV screening test, *Treponema pallidum* particle agglutination assay (TPPA), and, if indicated, the Venereal Disease Research Laboratory (VDRL) test. Additionally, PCR testing for *Chlamydia trachomatis* and *Neisseria gonorrhoeae* was routinely performed using anal/rectal swabs, pharyngeal swabs, and first-void urine samples.

The following data were collected securely according to data protection regulations: Participant surveys, laboratory test results, consultation notes of physicians and peer-to-peer consultants, medical prescription data, and billing and insurance information. All participants received sexual health counseling from trained peer advisors associated with Hein & Fiete, a community-based organization in collaboration with our center. Anonymous counseling was not available at our site, which may have represented a barrier for some individuals. However, in nearly all cases, the costs associated with PrEP provision and STI testing were covered by the German statutory or private health insurance systems.

### Study participant surveys

After written informed consent, each participant was assigned a pseudonymous study ID and received a printed QR code linking to the REDCap® baseline survey. Participants were encouraged to complete the survey at their convenience after their consultation. If the initial survey was completed at least six months prior, participants were asked to complete a follow-up survey. The questionnaires had a modular design allowed for tailored follow-up questions based on participants' initial responses.

The surveys covered: visit reason and referral source; sociodemographics, health-care utilization and barriers; prior STI/HIV testing and STI diagnoses with site; sexual behavior (frequency, partner numbers, oral/anal sex frequency, group sex, transactional sex, condomless anal sex by partner group); PrEP knowledge, use and regimen, pauses and side effects, information sources, motivations, preferences for future modalities, and partner selection by HIV/PrEP status; PrEP-related openness and stigma; substance use in sexual contexts (substances, routes, sharing of equipment); and vaccination status; sexual relationships and well-being. Item development was informed by an extensive review of the published literature and public-health reports and reviewed for face/content validity by infectious-disease clinicians and peer counselors in collaboration with the community partner Hein & Fiete prior to deployment; questions were purposefully designed to address documented data gaps in the German PrEP context. The last section comprised a simplified set of items adapted from the Multidimensional Sexuality Questionnaire [24, 25]; data from this section were not used in the present analyses.

Surveys were pseudonymized at first contact: each participant received a study ID and a REDCap® link/QR code. Clinical, laboratory, and counseling data required for analyses were linked via this pseudonymous ID and entered into the REDCap database by trained staff. The survey file and database operate offline, with access restricted to authorized study personnel, as stated in the questionnaire preface.

### Statistical analysis

Only participants who had received at least one PrEP prescription were included in the analysis. Prescriptions for HIV post-exposure prophylaxis (PEP) were identified (by indication and regimen combination) and excluded from PrEP analyses. Population characteristics were summarized using standard descriptive statistics.

Adherence was derived from routine care data rather than self-report. For each participant, we estimated PrEP coverage episodes for daily users using prescription records: each episode starts by prescription date and ends by end of pill coverage calculated from the dispensed quantity. An new prescription after that date indicates a new episode (Suppl. Figure 8). For each participant we summed covered days across all episodes to obtain days on PrEP, and defined the follow-up period from first prescription to the last coverage end (or censoring at September 19, 2024). Days on PrEP per person-year were calculated as (total covered days) ÷ (days of follow-up period/365.25). Pauses were defined as gaps ≥ 30 days between consecutive daily episodes, we also recorded documented reasons from clinical notes where available. Discontinuation was defined, for daily PrEP users, as no clinic visit after the expected end of pill coverage plus a 30-day grace period. We did not apply a fixed refill/visit gap because dispensing can be intentionally non-standard in routine care (e.g., advance supplies for extended travel, early refills, bridging prescriptions). For event-driven users and participants who switched between modalities, discontinuation was defined as no clinic visit within 180 days after the last prescription plus pill storage, before the analysis reference date (September 19, 2024). Reasons for discontinuation and pauses were ascertained through structured review of clinical records. Each discontinuation was classified as a documented planned stop or loss to follow-up (LTFU); when recorded, the stated reason was abstracted and categorized.

Laboratory-confirmed STI events were counted over follow-up and expressed as events per 100 person-years. Ninety-five percent confidence intervals were calculated using exact Poisson method providing appropriate coverage in strata with low event counts. We used Kaplan–Meier estimator to assess retention over time. For substance use analyses and correlations to STI exposures were summarized at the participant level: a binary “any sexualized substance use” indicator was set to positive if endorsed in either baseline or follow-up survey, and a “frequency category” (never/sometimes/often) was set to the higher (more frequent) in case of differing multiple responses.

Exploratory associations were evaluated using univariate logistic regression models yielding crude odds ratios (ORs) with 95% confidence intervals. Outcomes included dichotomized indicators of STI rate (above the cohort median), group sex at least monthly, illicit drug use (frequency of use not included) and substance use categories and interest in long-acting PrEP. We additionally fit a multivariable negative binomial regression (denominator: participants completed survey and had > 2 prescriptions) with a log–person-years offset to assess the association between interest in LA-PrEP and STI counts (adjusted for age, testing frequency, PrEP modality, illicit drug use, and group sex). Another multivariable negative binomial model (denominator: participants > 2 prescriptions) the association between age and STI counts (adjusted for PrEP, calendar, laboratory-visit intensity, number of PrEP phases, and baseline questionnaire completion). To assess trends in PrEP uptake during the COVID-19 and mpox pandemics a univariate linear regression model was fitted for each period to predict the proportion of active users over time**.** For renal function, changes in eGFR were evaluated using the Wilcoxon signed-rank test and a mixed-effects model to account for repeated measurements. The study design flowchart was created using draw.io. All other figures and all statistical analyses were performed using Python (Jupyter Notebook®).

### Ethical considerations

This study was approved by the Ethics Committee of the Medical Chamber of Hamburg (*Ethik-Kommission der Ärztekammer Hamburg;* Project Number PV7127). Written informed consent was obtained from all participants prior to enrolment. Procedures adhered to the ethical standards of institutional and national research committees and the Declaration of Helsinki (1964) with its subsequent amendments. Data collection and storage were pseudonymized and conducted in accordance with applicable local data protection regulations and the EU General Data Protection Regulation (GDPR).

## Results

**Fig. 1:**
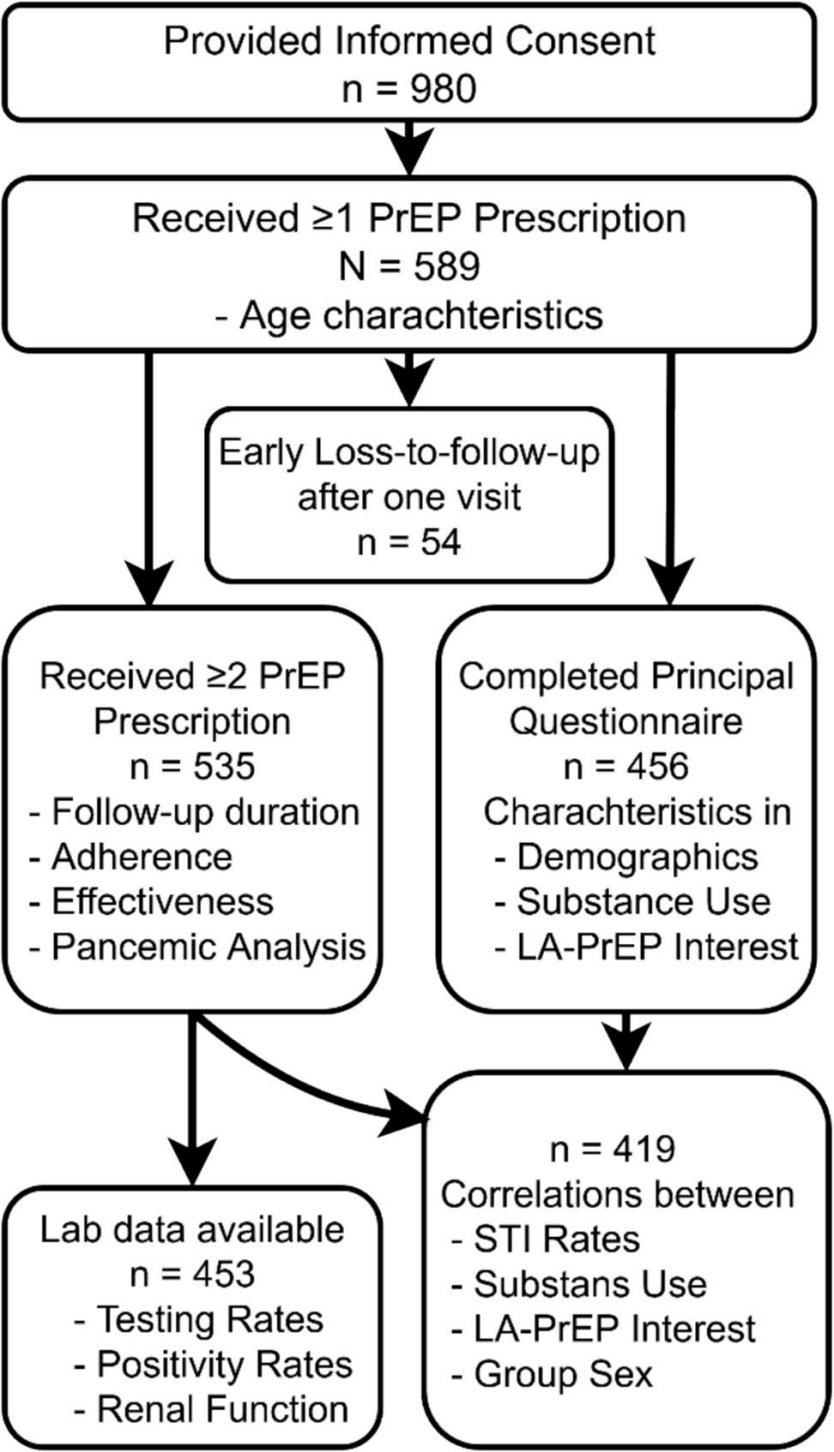
Study Design

Out of the total 980 participants who provided informed consent, only 589 received at least one prescription for Emtricitabine/Tenofovir between November 2019 and September 2024 and were therefore considered PrEP users and included in the detailed analysis as depicted in the study flow chart (Fig. 1). The remaining participants primarily presented for STI screening or counseling but did not initiate PrEP.

### Study population and characteristics

The median age of 589 participants, who received at least one TDF/FTC prescription was 32 years (range 18–70; Suppl. Figure 1) by first prescription. Of the 456 participants who completed the questionnaire, 97.1% identified as male, 83.7% as homosexual, and 81.8% were born in Germany. The majority had either a university or college degree (47.0%) or a high school diploma (24.8%), and most were in an open relationship (42.4%) or single (44.5%). (Table 1). Only 4.7% of the participants who completed the principal questionnaire identified as People of color.

**Table 1:** Characteristics of the study population

	Count (%)
Age (years) ^‡^	*N* = *589*
18–24	74 (12.6)
25–29	131 (22.3)
30–39	214 (36.4)
≥ 40	169 (28.7)
Gender ^†^	*N* = *456*
No Answer/Missing	40
Male	404 (97.1)
Female	4 (1.0)
Transgender	5 (1.2)
Non-binary	2 (0.5)
Other	1 (0.2)
Sexual Orientation ^†^	*N* = *456*
No Answer/Missing	40
Homosexual	348 (83.7)
Bisexual	37 (8.9)
Queer	10 (2.4)
Heterosexual	9 (2.2)
Other	5 (1.2)
Unsure / Not Clear	7 (1.7)
Relationship status * ^†^	*N* = *456*
No Answer/Missing	166*
Single / No Committed Relationship	129 (44.5)
Open Relationship	123 (42.4)
Monogamous Relationship	29 (10.0)
Polyamory	6 (2.1)
Other	3 (1.0)
Educational Level ^†^	*N* = *456*
No Answer/Missing	41
University / College Degree	195 (47.0)
High School Diploma (Abitur)	103 (24.8)
Apprenticeship / Vocational Training	66 (15.9)
Secondary / Middle / Elementary School Diploma	39 (9.4)
Doctorate (PhD)	11 (2.7)
No School Diploma	1 (0.2)
Region of birth ^†^	*N* = *456*
No Answer/Missing	41
Germany	339 (81.8)
European Union	27 (6.5)
Europe (non-EU)	15 (3.6)
Middle East	11 (2.6)
South America	8 (1.9)
East Asia	5 (1.2)
North America	5 (1.2)
Central Asia	2 (0.5)
South Asia	1 (0.2)
Southeast Asia	1 (0.2)
Sub-Saharan Africa	1 (0.2)

Age (‡) derives from clinical records; Age groups were calculated based on the participant’s age at PrEP initiation; all other characteristics (†) from the principal questionnaire; Educational level item (*) was revised after initiation; “No Answer/Missing counts are shown for transparency but are excluded from percentage denominators; Percentages are calculated among available answers for each item (available-case; n = N − missing) and are rounded to one decimal place

**Fig. 2:**
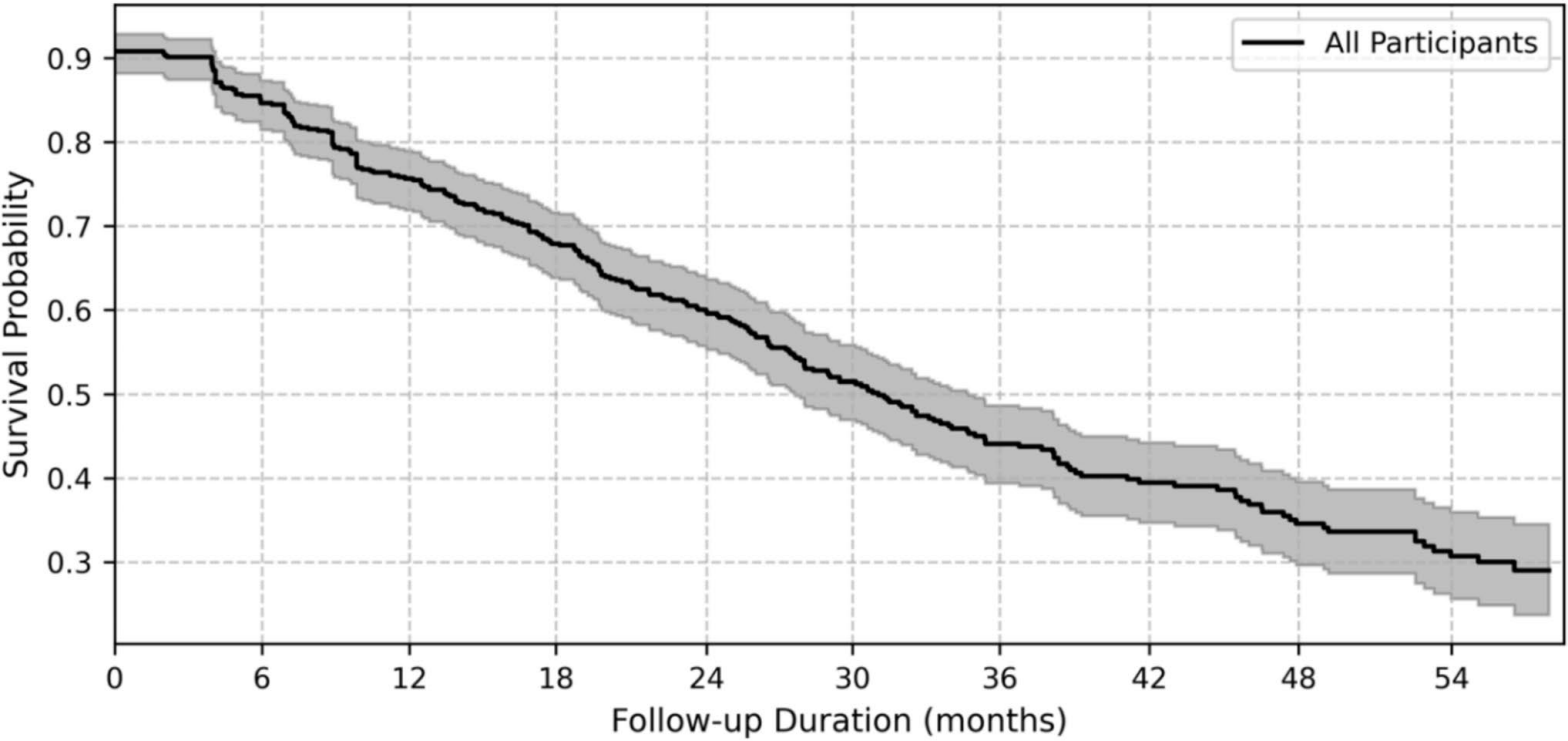
Kaplan–Meier estimate of PrEP follow-up duration. The survival curve shows the probability of participants remaining active on PrEP over time. The shaded area represents the 95% confidence interval. Participants were censored at the end of their follow-up or when LTFU (n = 535)

Participants with only one PrEP prescription (n = 54) were labeled as early LTFU. Among participants with at least 2 prescriptions (n = 535), the overall mean follow-up duration was 102.3 weeks (SD: 75.7), with an IQR of 38.6 to 151.4 weeks. Participants who remained active until the end of the observation period had an average follow-up of 133.9 weeks (SD: 76.9; IQR: 76–194.3). LTFU participants had a mean follow-up duration of 73 weeks (SD: 62; IQR: 18.9–116) (Fig. 2 and Suppl. Fig. 2).

### Adherence in PrEP use

Among all 589 participants, they attended 4828 visits during the follow-up period, with an average of 8.2 visits per participant (SD = 5.9, IQR: 3–11). Among participants with at least 2 prescriptions (n = 535), the total follow-up time was 1189.5 person-years, corresponding to a mean of 2.22 years per participant (SD = 1.37, IQR: 1.09–3.20). At the reference date (September 19, 2024), 313 participants (53.1%) remained active on PrEP. In total, 65 participants (11.0%) had a documented planned discontinuation, and 155 participants (26.3%) were classified as LTFU. 54 participants (9.2%) were lost to follow-up after their initial prescription. The overall mean quarterly dropout rate was 3.56% (IQR 2.65%–4.83%).

Among those who discontinued PrEP with documented reasons, the most common reasons cited were entering a partnership (n = 21), changing medical center (n = 12), and moving away (n = 8). Other individually reported reasons included lack of perceived need, gastrointestinal side effects, mental health issues, financial barriers, and planned international stays.

**Fig. 3:**
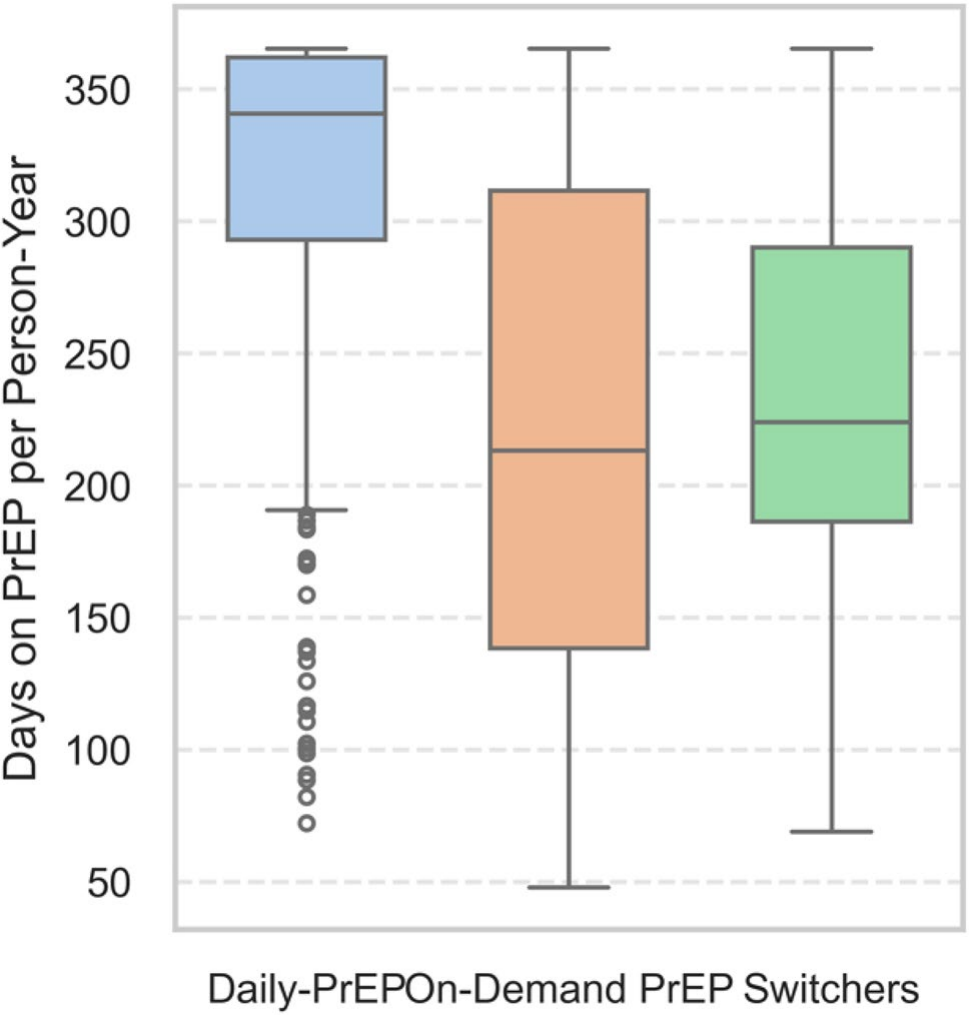
Days on PrEP per PY by PrEP usage group. Boxplots show the distribution of days on PrEP per PY among daily users (n = 421), on-demand users (n = 79), and switchers (n = 29), defined as participants alternating between daily and on-demand PrEP modalities during analysis period. Horizontal lines represent medians; boxes show interquartile ranges; whiskers represent 1.5 × the interquartile range; individual points indicate outliers

Among Daily-PrEP users (n = 421), the mean number of days on PrEP per PY was 315.1 days, with a median of 340.7 days (IQR: 293.0–361.9 days). For on-demand PrEP users (n = 79), the mean was 218.8 days, with a median of 213.1 days (IQR: 138.4–311.6 days). Among switchers, defined as participants alternating between daily and on-demand PrEP modalities (n = 29), the mean days on PrEP per PY was 238.4 days, with a median of 223.9 days (IQR: 186.3–290.0 days) (Fig. 3).

Among daily-PrEP users a total of 370 pauses (defined as pill shortage ≥ 30 days) were recorded. The mean number of pauses per participant was 0.87, with a median of 0 (range: 0–5). Average cumulative pauses were 41.7 days per PY (range 0–288 days). Reasons for pausing PrEP were documented for 67 pauses, whereas 149 records lacked a specified reason. Among documented reasons, the most frequently cited were no perceived need for PrEP (n = 15), COVID-19-related circumstances (n = 11), changes in partnership status (n = 9), scheduling difficulties (n = 4), and travel or temporary relocation (n = 4). Less common reasons included medical conditions (e.g., illnesses, surgeries, chemotherapy), occupational demands, insurance barriers, and side effects. Overall, the majority of pauses appeared to be driven by personal or logistical considerations rather than adverse medical events.

Given this adherence profile, we subsequently examined the actual effectiveness of PrEP in preventing HIV infections within this cohort.

### Effectiveness of PrEP in HIV prevention

Only two new HIV infections were identified among participants. One participant tested HIV-positive prior to initiating PrEP, while the second infection occurred 1148 days after initiation and after the analysis reference date. This participant had discontinued PrEP 738 days prior to diagnosis due to gastrointestinal intolerance (Suppl. Table 1 and Suppl. Figure 7). Both individuals reported no PrEP use within 30 days prior to their estimated HIV exposure.

As no HIV infections occurred during active PrEP use within the analysis period, the observed HIV incidence rate was 0 per 100 person-years. Although the absence of a control group limits a direct estimation of effectiveness, the subsequent analysis of sexual behaviors and STI rates provides indirect evidence regarding PrEP's real-world impact.

### Sexual behaviors characteristics

A total of 631 STIs were diagnosed in 263 participants during the observation period. Participants with only one PrEP visit (n = 54) were excluded from the person-years analysis due to insufficient follow-up time. Among this subgroup, 17 STI diagnoses were observed, most commonly *Neisseria gonorrhoeae* (8 cases), *Chlamydia trachomatis* (4 cases), and syphilis (2 cases).

Among participants with more than two PrEP visits, 613 STI diagnoses were recorded (51.5/100 PY; 95% CI 47.5–55.8); pathogen-specific counts and rates are shown in Table 2.

**Table 2:** STI Counts and Rates (Participants > 2 Visits)

	Count (%)	Cases/100 PY (95% CI)
Overall	613	51.5 (47.5–55.8)
*Chlamydia trachomatis*	251 (40.9)	21.1 (18.6–23.9)
*Neisseria gonorrhoeae*	224 (36.5)	18.8 (16.4–21.5)
Syphilis	65 (10.6)	5.5 (4.2–7.0)
*Condylomata acuminata*	34 (5.5)	2.9 (2.0–4.0)
*Herpes genitalis*	12 (2.0)	1.0 (0.5–1.8)
Mpox	6 (1.0)	0.5 (0.2–1.1)
*Mycoplasma*	4 (0.7)	0.3 (0.1–0.9)
Hepatitis C	2 (0.3)	0.2 (0.0–0.6)
Others	15 (2.4)	1.3 (0.7–2.1)

This table summarizes sexually transmitted infection (STI) diagnoses among participants with more than two PrEP follow-up visits during the analysis period. Percentages represent the proportion of each STI relative to the total number of documented cases. Incidence rates are presented as cases per 100 PY, with 95% confidence intervals (CI) calculated assuming a Poisson distribution. "Others" included scabies, genital mycoses, and streptococcal dermatitis. Seventeen STI diagnoses were from participants with ≤ 2 visits and were excluded from the person-years analysis

To clarify the variation in STI risk among subgroups, we estimated individual STI acquisition rates per 100 PY (PY) based on each participant's follow-up duration. After excluding rates > 400/100PY due to very short follow-up period and stratified by PrEP modality, the Exact Poisson overall STI Rates/100PY were 52.4 /100 PY (95% CI: 47.8–57.4, n = 421) for daily PrEP users, 38.9/100 PY (95% CI: 30.1– 49.5, n = 79) for event-driven users, and 54.8 /100 PY (95% CI: 40.9 – 71.8, n = 29) for switchers (those alternating between modalities) (Suppl. Figure 3).

Using a univariate logistic regression with the outcome *‘high STI rate’*—defined as a participant’s overall STI incidence exceeding the cohort median (events per 100 person-years)—event-driven PrEP users had lower odds than daily users (OR = 0.50, 95% CI 0.30–0.85; p = 0.010). Reporting group sex ≥ monthly was associated with higher odds (OR = 1.92, 95% CI 1.10–3.34; p = 0.021).

**Fig. 4:**
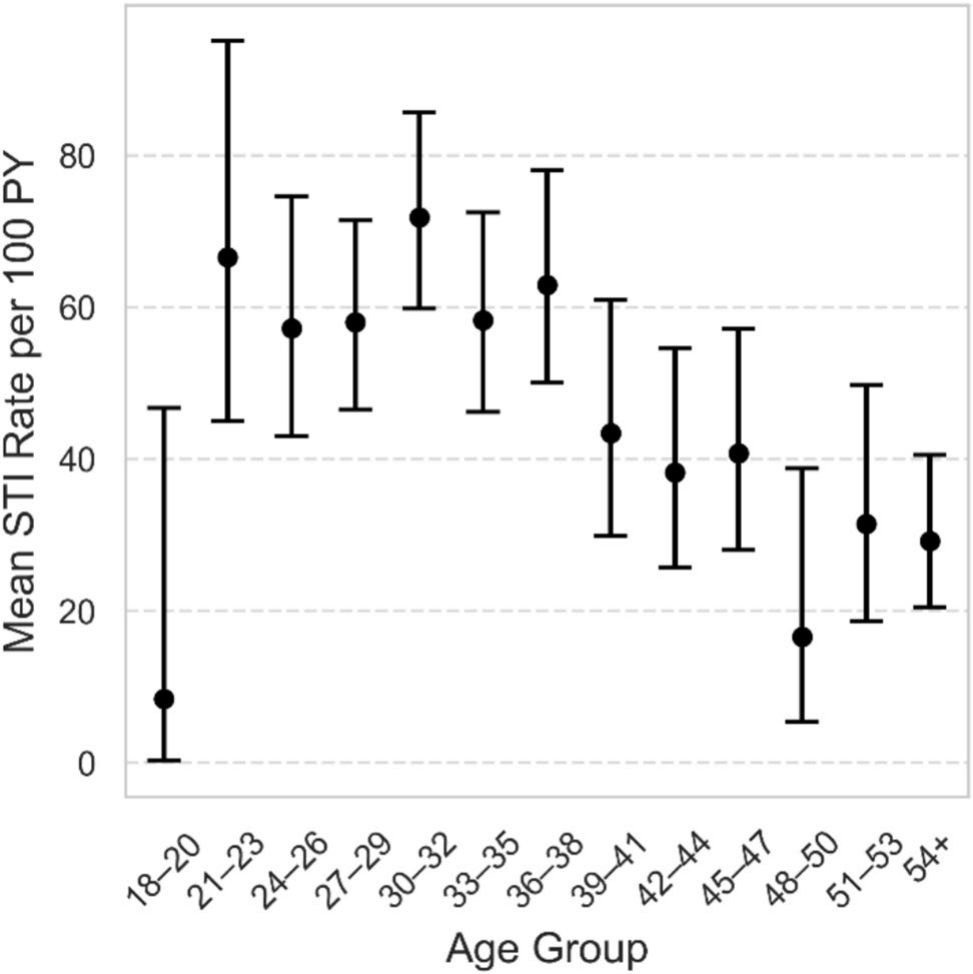
Mean STI incidence rate per 100 PY, stratified by age group. Age groups are defined by each participant’s mean age during follow-up (midpoint between first and last/dropout). Points show the group-level incidence rate per 100 person-years; vertical bars are exact 95% Poisson confidence intervals

STI incidence varied by age group, with the highest mean rates observed among participants aged 30–32 and 27–29 years. Participants aged 18–20 showed the lowest rates of STIs. Overall, STI rates tended to peak in individuals in their early to mid-30s, followed by a decline in older age groups (Fig. 4). A multivariant negative binominal model showed a significant negative association between age and overall STI rates (Incidence Rate Ratio per 10 Years = 0.82; 0.72–0.94, p = 0.00448). An exploratory unadjusted logistic model shows that participants aged over 50 years had lower rate of *Neisseria* *gonorrhoeae* (OR = 0.35, 95% CI: 0.15–0.80, p = 0.013). An unexpected finding by this model was the isolated increase in rates of *Chlamydia trachomatis* infection among participants aged 25–30 years (OR = 1.94, 95% CI: 1.21–3.11, p = 0.006). A weak but significant positive correlation was found between STI testing rates and STI diagnosis rates (r = 0.34). Each additional test per 100 PY was associated with an increase of 0.11 STI cases per 100 PY (95% CI: 0.08–0.13).

### Impact of COVID-19 Pandemic on PrEP use patterns

In Hamburg, the first COVID-19 lockdown began on March 2020, with subsequent restrictive measures imposed during a second lockdown in November 2020 and a stricter “hard” lockdown starting December 2020. Despite these public health interventions, the mean number of active PrEP users continued to rise steadily from late 2019 onward, without significant shifts in growth rates during or immediately after the lockdown periods. A noticeable slowdown in new PrEP initiations occurred later in mid-2023, primarily due to capacity constraints at our clinic (Suppl. Figure 4).

**Fig. 5:**
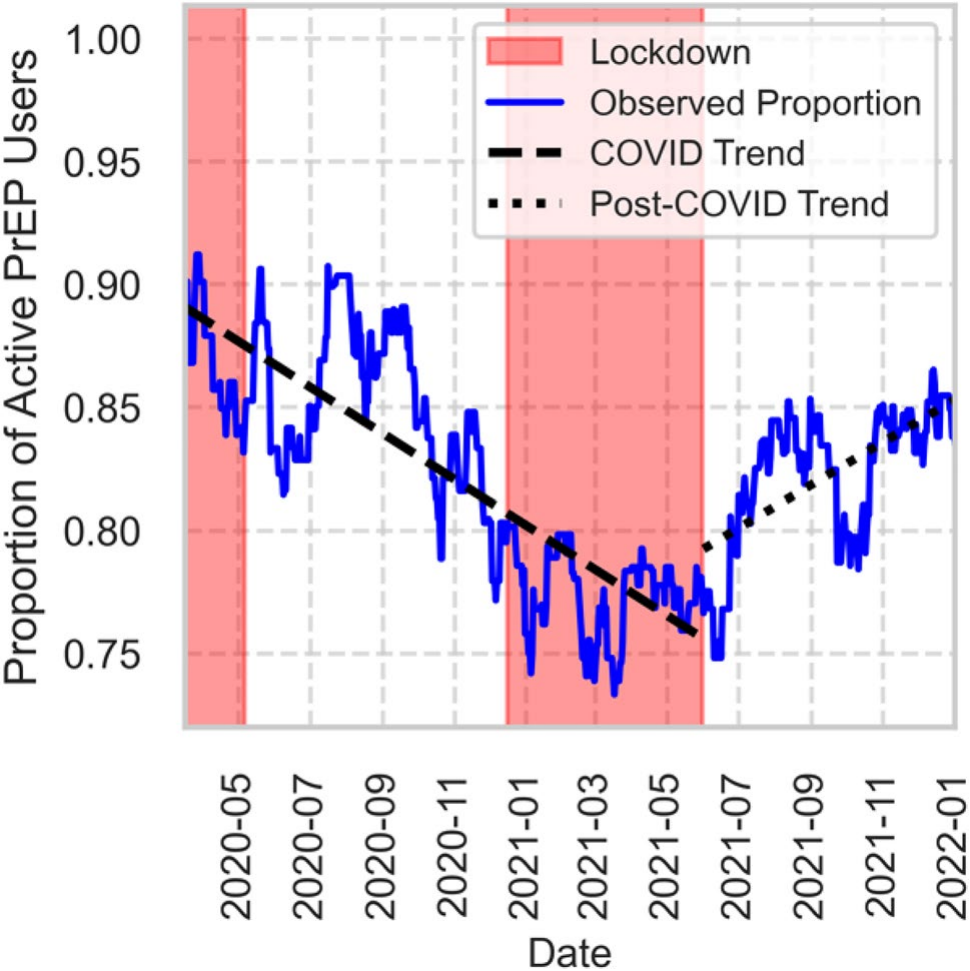
Proportion of active PrEP users during and after the COVID-19 pandemic. The solid line shows the share of daily PrEP users who maintained sufficient pill availability, expressed as a proportion of all non-LTFU participants. Shaded regions represent official COVID-19 lockdown periods in Hamburg. Dashed and dotted lines indicate linear regression trend estimates during the COVID and post-COVID periods, respectively

Among the active PrEP participants, no changes in testing rates or positivity rates were registered during the pandemic (Suppl. Figure 5 and Suppl. Figure 6). Among daily PrEP users, the proportion actively engaged in PrEP use decreased during the COVID-19 pandemic (slope = − 0.0003 per day, R^2^ = 0.67, p < 0.0001). Following the end of restrictions, a modest recovery was observed (slope = + 0.0003 per day, R^2^ = 0.40, p < 0.001) (Fig. 5). A stronger but shorter effect was registered in the second half of the mpox pandemic (slope = − 0.0013 per day, R^2^ = 0.89, p < 0.0001).

### Substance use and correlations to STDs

Of 456 participants who completed the principal questionnaire, only 388 (85%) provided a response to “*When you have sex, do alcohol or other substances play a role?*” (Table 3).

**Table 3:** Sexualized Substance Use

	Sometimes	Often	Total
Alcohol	164 (42.3)	44 (11.3)	208 (53.6)
Poppers	86 (22.2)	35 (9.0)	121 (31.2)
Cannabis	39 (10.1)	14 (3.6)	53 (13.7)
Potency meds	26 (6.7)	17 (4.4)	43 (11.1)
Ecstasy/ MDMA	18 (4.6)	9 (2.3)	27 (7.0)
Cocaine	11 (2.8)	11 (2.8)	22 (5.7)
Amphetamine	13 (3.4)	7 (1.8)	20 (5.2)
GBL/GHB	5 (1.3)	7 (1.8)	12 (3.1)
Ketamine	2 (0.5)	8 (2.1)	10 (2.6)
Crystal Meth	0	3 (0.8)	3 (0.8)
Others	1 (0.3)	1 (0.3)	2 (0.5)
Crack/Heroin	0	0	0

Percentages are calculated among respondents to the item (available-case; n = 388) from all participants who completed the principal questionnaire (N = 456). Participants with no response (n = 68; 15% of N) were excluded from denominators. “Total” is the pooled proportion reporting either Sometimes or Often for each substance. Multiple responses were possible. “Potency meds” denotes erectile dysfunction medications. “GBL/GHB” includes gamma-butyrolactone and gamma-hydroxybutyric acid. “Others” includes mephedrone and 3-methylmethcathinone (3-MMC). When participants completed more than one questionnaire, discrepant non-missing responses were reconciled by retaining the highest category (Never < Sometimes < Often) reflecting peak reported use

**Fig. 6:**
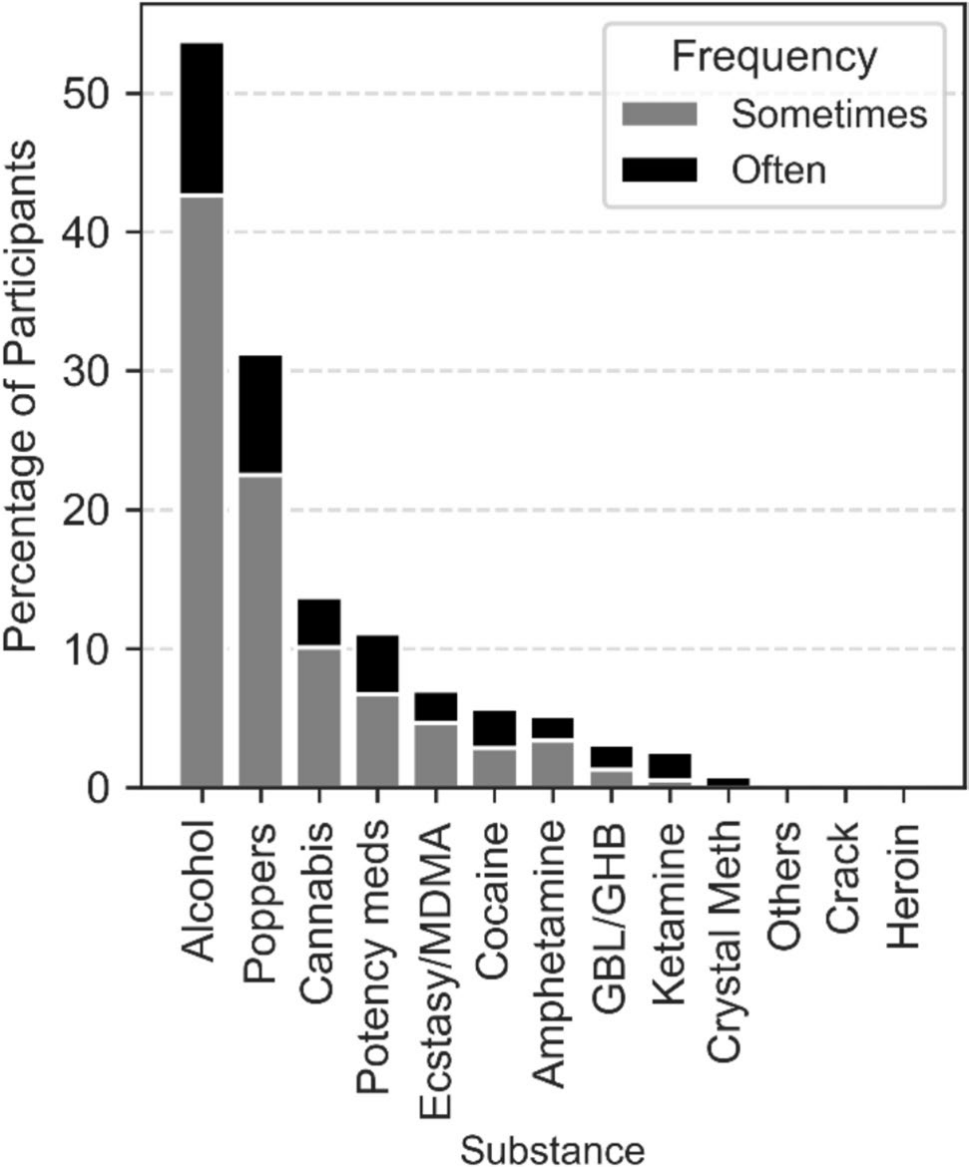
Frequency of sexualized substance use among PrEP users (available-case; n = 388) Participants with no response to the item (n = 68; 15% of N = 456) were excluded from denominators. Stacked bar chart showing the percentage of participants who reported using substances in sexualized context. Frequencies are categorized as "Sometimes" (gray) and "Often" (black). Percentages are based on all participants who responded to substance use questions, multiple responses possible

Higher-risk substances were grouped into an 'illicit substances' category—including ecstasy/MDMA, cocaine, GBL/GHB, ketamine, crystal meth, and others—revealing that 59 participants (14.3%) reported any use. Most reported occasional use, while only a minority indicated frequent consumption. No participants reported the use of crack or heroin. (Fig. 6).

This analysis highlights a substantial prevalence of substance use, particularly of substances commonly associated with “chemsex” within the cohort [18, 19]. Using a dichotomized variable for any substance use (pooled for “Sometimes” and “Often” and considering peak reported use, when differing use reported in recurring questionnaires) the frequency of group sex did not differ significantly between substance users and non-users (p = 0.2402) and the use of these substances was not associated with higher overall STI rates compared to non-consumers. Notably, poppers use was the only drug associated with an increased risk of *Neisseria gonorrhoeae* infection (OR = 1.63, 95% CI: 1.03–2.56, p = 0.035), while no significant associations were found for *Chlamydia trachomatis* infection or syphilis. MSM under 40 years old who indicated taking erectile meds (sometimes and often) used more frequently illicit substances (53% vs 12%, OR 8.25, p < 0.0001). All MSM under 40 reporting frequent use (“Often”) of erectile dysfunction medications also reported illicit substance use.

### Interest in LA-PrEP

**Fig. 7:**
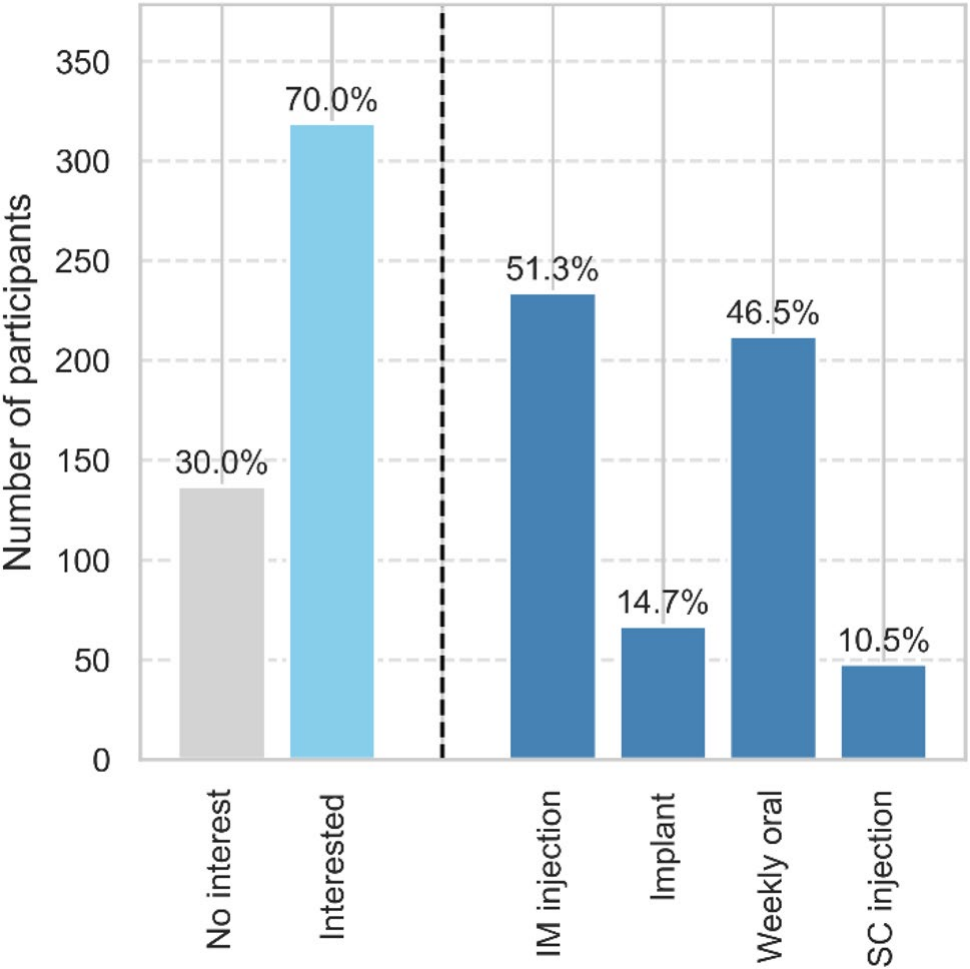
Participant interest in and preferences for LA-PrEP. Bars show counts; values above bars show percent of available cases (n = 456). Left panel contrasts “No interest” vs “Interested (any option)”; right panel displays preferences for specific modalities (IM injection, implant, weekly oral, SC injection). Preferences are not mutually exclusive; modality percentages use the same denominator and can sum to > 100%; No answer/missing = 133 of 589; *IM* intramuscular; *SC* subcutaneous

Among participants, interest in LA-PrEP formulations was high, with intramuscular injectable options being the most preferred (n = 234), followed by weekly oral tablets (n = 212), implants (n = 67), and subcutaneous injections (n = 48). (Fig. 7).

In a multivariate negative binomial model interest in LA-PrEP was not associated with higher STI incidence (p = 0.95). An exploratory unadjusted logistic model shows that participants expressing interest in LA-PrEP had higher odds of reported illicit substance use (OR = 5.54, 95% CI: 1.95–15.73, p = 0.001). Adherence, measured as days on PrEP per PY, did not significantly differ between participants interested in long-acting PrEP and those not interested (mean difference: 3.2 days/PY, p = 0.67, 95% CI: –11.8 to 18.1).

### Renal function assessment

**Fig. 8:**
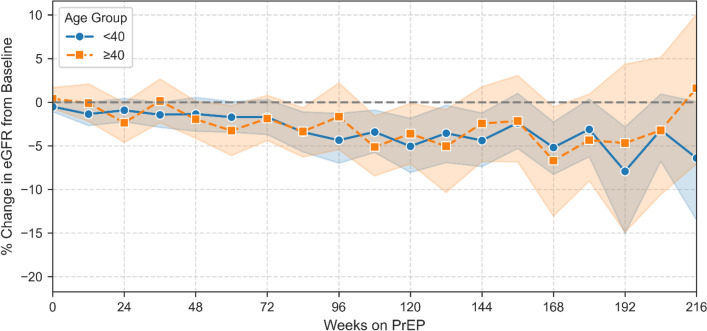
Change in renal function (eGFR) over time on PrEP, stratified by age group (n = 421). The plot shows the mean percentage change in estimated glomerular filtration rate (eGFR) from baseline, binned by 12-week intervals. Lines represent age groups 18–39 and 40 + , with 95% confidence intervals. The dashed horizontal line at 0% represents no change from baseline

The median eGFR at the last available measurement was 100 mL/min/1.73 m^2^ (IQR: 89–113), reflecting a median decrease of 4 mL/min/1.73 m^2^ compared to baseline (p < 0.001, Wilcoxon signed-rank test). In a mixed-effects model that accounted for repeated laboratory measures per participant, age showed a strong inverse association with eGFR (β = − 0.95, p < 0.001), consistent with physiological decline in renal function over time. However, cumulative PrEP exposure (total prescribed pills or intensity relative to follow-up) was not significantly associated with eGFR, suggesting no evidence of adverse renal effects related to PrEP use in this cohort. (Fig. 8).

Overall, FTC/TDF-based PrEP was well tolerated with minimal impact on renal function over the observation period.

## Discussion

This prospective observational study presents real-world data from a large German PrEP cohort and provides one of the few available sources of pseudonymized, participant-linked longitudinal data in this field whereas most existing studies in Germany rely on anonymous surveys or provider-reported information [6, 26–28]. The cohort consisted predominantly of young, well-educated MSM. This participant profile is consistent with findings from other German and European studies [29–32]. Although most participants were born in Germany, roughly one quarter reported being born abroad. In Hamburg, about 41% of the population have a migration background; however, the official definition (nationality at birth of the individual or at least one parent) does not align with our survey variables (countries of birth only), limiting direct comparability [33]. Nevertheless, based on surveillance data, Germany's HIV epidemic disproportionately affects foreign-born individuals [34], aligning with the observed underrepresentation in our study. While the majority of PrEP users are MSM, the proportion of women in our cohort was negligible. Although German PrEP guidelines are not restricted to MSM, uptake among women in our sample was minimal, while recent national estimates suggest that ~ 21% of new HIV infections in Germany occur in women. These findings align with further studies [6, 27] and indicates access barriers requiring targeted interventions to achieve equitable prevention impact. To assess these gaps more robustly, multilingual, multicenter surveys targeting underrepresented populations are needed.

### Effectiveness and adherence

Assessing PrEP adherence is inherently challenging, especially since oral PrEP can remain highly effective even when not taken daily—despite most guidelines recommending daily use as the standard regimen [35]. PrEP coverage per PY in our cohort was relatively high, particularly among daily users. We observed moderate rates of PrEP discontinuation and temporary interruptions. When available, the reported reasons showed predominantly changes in life situation with reported lower risk behavior (partnership status, moving, illness, COVID-19 pandemic).

Furthermore, PrEP in our cohort during the observation period was highly effective, although we observed high incidence of sexually transmitted infections—especially *Chlamydia trachomatis* and *Neisseria gonorrhoeae* —indicating substantial ongoing sexual risk and, consequently, potential HIV exposure. Despite this, no HIV infections occurred during the analysis period. The single documented HIV infection occurred long after the individual's last PrEP prescription and after the completion of data collection, thus it was not included in the statistical analysis. This infection was associated with non-adherence reportedly due to gastrointestinal intolerance.

Collectively, these findings indicate that individual variations in risk profiles and life circumstances significantly influence PrEP uptake. This underscores the importance of offering multiple patient-centered PrEP modalities with adaptable follow-up intervals to optimize retention.

### STI burden and substance use correlates

The observed association between poppers use and increased risk of *Neisseria gonorrhoeae* infection, but not *Chlamydia trachomatis*, may reflect physiological effects of poppers on mucosal integrity or due to behavioral patterns [36]. The effect of poppers could lead to increased duration and intensity of the sexual activity lowering the transmission barrier of *N. gonorrhoeae*. A similar finding was reported in a cohort in Amsterdam, only in HIV-negative MSM [37]. Other studies showed immunomodulating effects in other drugs, used in sexualized context [38, 39].

In contrast to *Neisseria gonorrhoeae* infection, younger participants in our cohort showed a higher risk of *Chlamydia trachomatis* infection. To our knowledge, this age-specific pattern has not been previously reported. Possible explanations include detection bias, age-assortative sexual networks, or differences in acquired immunity among age groups. Notably, in our study, the use of *chemsex*-related substances and other illicit substances was not associated with significantly higher STI rates compared to non-users.

### Impact of the COVID-19 pandemic

During the COVID-19 pandemic, the rate of active daily PrEP users in our cohort declined, followed by a gradual increase in the post-pandemic period. A similar but shorter pattern was observed during the mpox outbreak. Interestingly, despite the temporary decline in PrEP engagement, both STI testing rates and STI positivity remained stable. This contrast suggests that while some participants paused PrEP due to a perceived lower risk, many others continued to engage in high-risk sexual behavior. Overall, STI transmission patterns in the cohort appeared largely unaffected by pandemic-related public health restrictions. Similar findings have been reported by other sexual health centers [30].

### Interest in long-acting PrEP

As long-acting PrEP options are currently either not available or not covered by health insurance in Germany, access to such modalities remains practically unavailable. Despite higher effectiveness of LA-PrEP [11, 12], participants in our cohort expressed a high level of interest in these options, especially among individuals with a history of substance use. This association suggests that long-acting formulations may be particularly appealing to individuals who experience difficulties maintaining adherence to daily or event-driven PrEP regimens [40].

### Renal function monitoring and simplified safety

Renal function remained stable in our cohort. A small but statistically significant median decline in eGFR was observed over the follow-up period. It is important to note that younger MSM participants in our cohort frequently reported the use of creatine supplements for muscle gain. This factor could not be addressed in the current analysis but is intended for further investigation.

Importantly, no association was found between cumulative PrEP exposure and changes in eGFR. These findings support the German-Austrian guideline recommendation for quarterly renal monitoring, while also aligning with international trends suggesting that less frequent monitoring may be safe in younger, otherwise healthy individuals [23].

### Limitations

Several limitations must be acknowledged. First, the observational design of this study precludes causal inference; Second, the lack of a formal control group limits our ability to directly estimate the effectiveness of PrEP in preventing HIV. Third, behavioral data were self-reported and subject to recall bias. Fourth, follow-up completeness varied, particularly among participants who discontinued PrEP after a single visit, which may have introduced bias in adherence or outcome estimates. Importantly, we had no access to HIV or STI outcomes after dropout; infections diagnosed elsewhere may have been missed. Fifth, the principal questionnaire was only available in German and voluntary, potentially limiting full participation from non-native speakers and introducing selection bias. Sixth, most association analyses were univariate and therefore subject to residual confounding; effect estimates should be interpreted as descriptive. Seventh, survey completion was asynchronous with testing, exposures (e.g., sexualized substance use) were summarized at the participant level (any/peak frequency across baseline/follow-up) and interest in LA-PrEP was available only at baseline; this may have introduced exposure misclassification. Lastly, the potential influence of creatine supplementation on eGFR measurements, especially among younger MSM could not be accounted for. Generalizability may also be limited, as participants were primarily MSM accessing care in an urban, community-based sexual health setting with structured support, which may not reflect all PrEP-eligible populations.

## Conclusion

Our data supports a flexible, person-centered PrEP model. Marked changes in use during COVID-19 and mpox, and variability in individual risk, argue for simplified switching between daily and event-driven regimens. Persistently high STI incidence justifies targeted intensification (e.g., for group sex involvement or frequent sexualized drug use) and de-escalation during stable low-exposure phases. High HIV prevention effectiveness and largely stable renal function support a risk-stratified de-escalation. Services should also incorporate LA-PrEP, advance dispensing, and multilingual access, alongside women- and migrant-inclusive outreach, to improve retention, equity, and progress toward national HIV-prevention goals.

## Supplementary Information

Below is the link to the electronic supplementary material.

Supplementary file1 (DOCX 17393 KB)

## Data Availability

The pseudonymised participant data underlying this study are stored on the secure, controlled-access server of the University Medical Center Hamburg-Eppendorf. Due to local-ethics restrictions, the dataset cannot be made publicly available. De-identified data can be shared for non-commercial, ethically approved research on reasonable request to the corresponding author and upon completion of a data-sharing agreement approved by the projects data sharing comithee.

## Abbreviation

HIV: Human immunodeficiency virus
WHO: World health organization
PrEP: Pre-exposure prophylaxis
TDF/FTC: Tenofovir disoproxil fumarate / emtricitabine
EMA: European medicines agency
MSM: Men who have sex with men
LA-PrEP: Long-acting pre-exposure prophylaxis
LTFU: Loss to follow-up
STI: Sexually transmitted infection
eGFR: Estimated glomerular filtration rate
DSGVO: Datenschutz-grundverordnung (german data protection regulation)
GDPR: General data protection regulation
SD: Standard deviation
IQR: Interquartile range
PY: Person-years
CI: Confidence interval
RKI: Robert koch institute
OR: Odds ratio
IM: Intramuscular
SC: Subcutaneous
PhD: Doctor of philosophy
GBL: Gamma-butyrolactone
GHB: Gamma-hydroxybutyrate
