# Supplementary material for: Five-Year German PrEP cohort reveals high HIV protection and persistent STI burden: implications for individualized and flexible prevention strategies
Source: Infection. 2025 Oct 25;54(1):339–52. doi: 10.1007/s15010-025-02667-w (PMC12864345; doi:10.1007/s15010-025-02667-w)
Supplement: Supplementary file 1 — Supplementary file1 (DOCX 17393 KB) [file 15010_2025_2667_MOESM1_ESM.docx]

**Supplementary Materials**

Five-Year German PrEP Cohort Reveals High HIV Protection and Persistent STI Burden: Implications for Individualized and Flexible Prevention Strategies

Maher Almahfoud, Lukas Weimann, Guido Schäfer, Till Koch, Hanna Matthews, Hanna M. Weichel, Friederike Hunstig, Marc Grenz, Robin R. Scheiter, Marylyn M. Addo, Julian Schulze zur Wiesch, Olaf Degen

**Suppl. Fig. 1** Distribution of age at PrEP initiation among participants (n = 589)

The histogram displays the frequency of individuals at each age from 18 to 68 years at the time of PrEP initiation. Age was calculated based on the date of PrEP start.


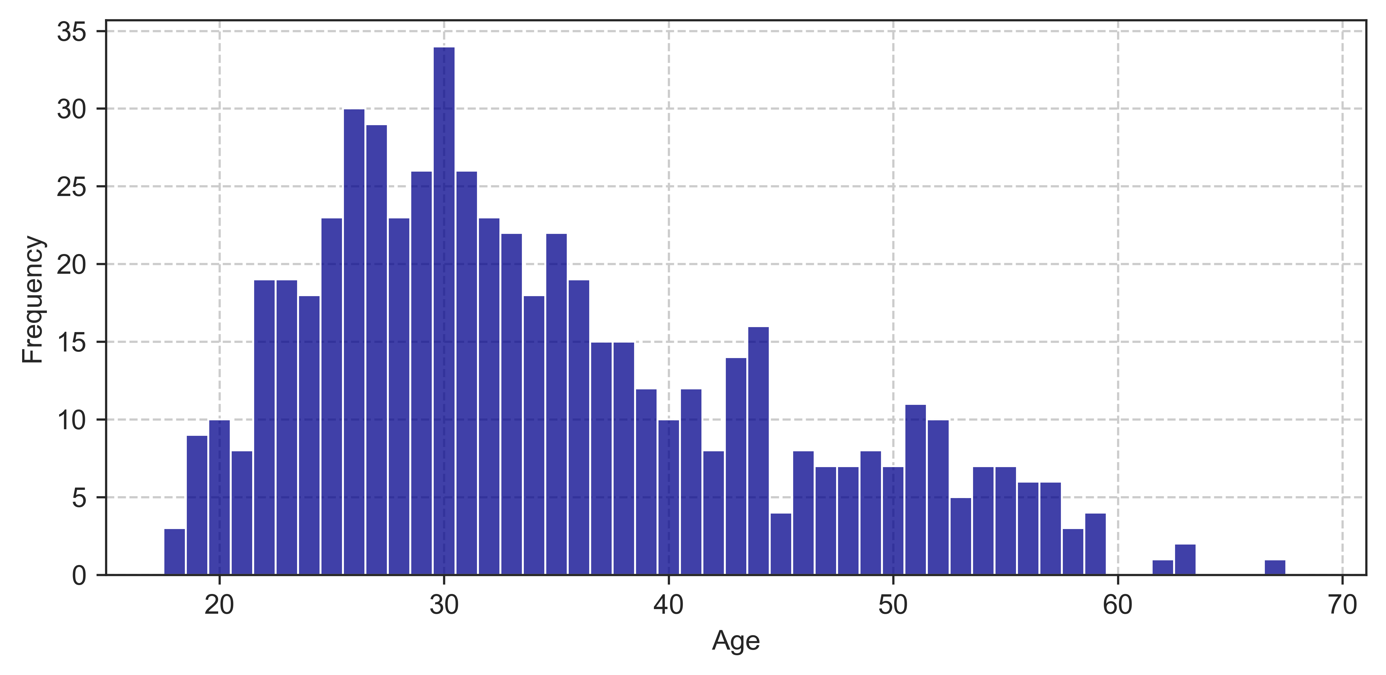


**Suppl. Fig. 2** Distribution of PrEP follow-up duration among participants.
data lines show the number of participants by follow-up duration in days, separated by follow-up status. The black indicates participants still active as of the reference date, and the gray lines shows participants lost to follow-up before the reference date (September 19, 2024)


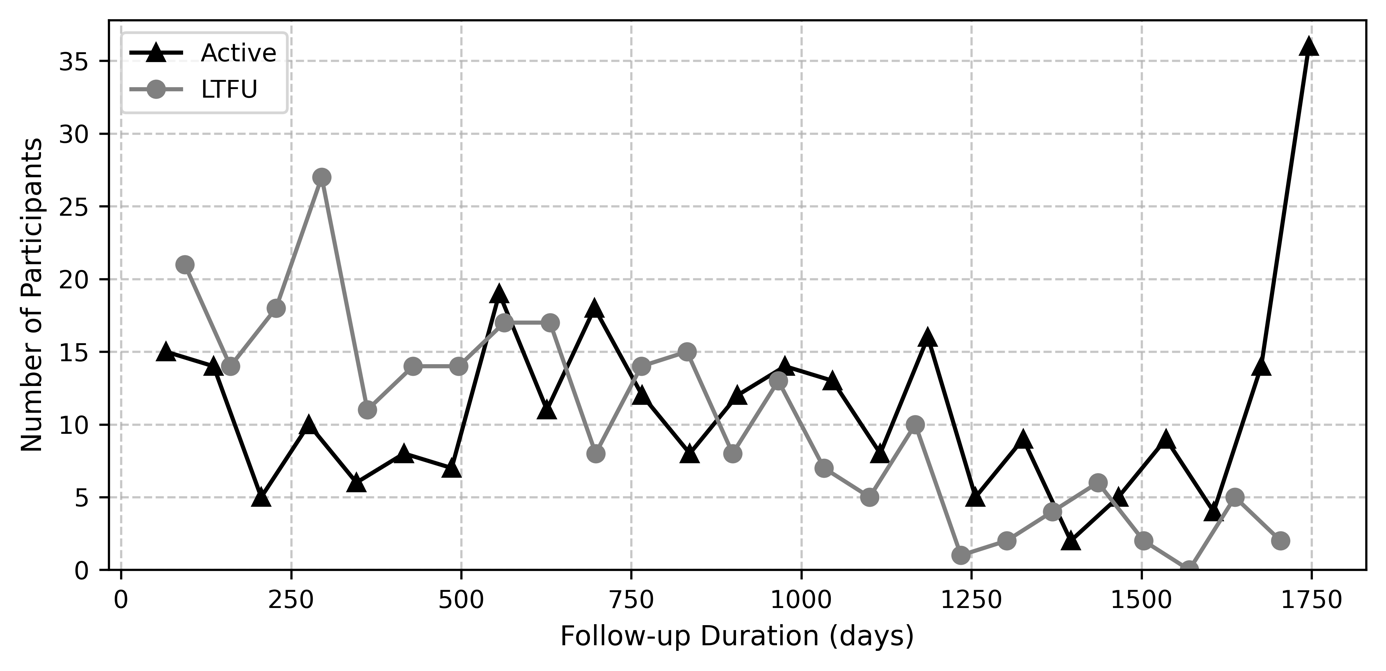


**Suppl. Fig. 3** STI rates by PrEP modality

Bars show group-level STI rates with exact 95% Poisson confidence intervals; individual participants are shown as jittered dots, and red “X” markers indicate excluded outliers (very short follow-up yielding extremely high rates; here defined as ≥400 per 100 PY). The y-axis is on a logarithmic scale. Rates are expressed per 100 person-years (PY).
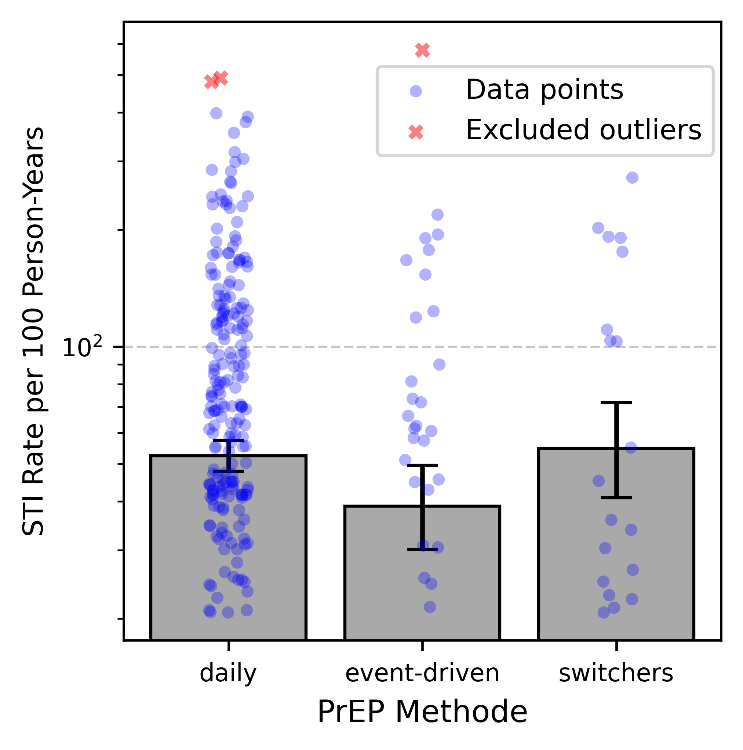


**Suppl. Fig. 4** Quarterly number of new PrEP participants (n=594)

Bars represent the number of individuals initiating PrEP in each calendar quarter. The shaded area highlights the period affected by the COVID-19 pandemic


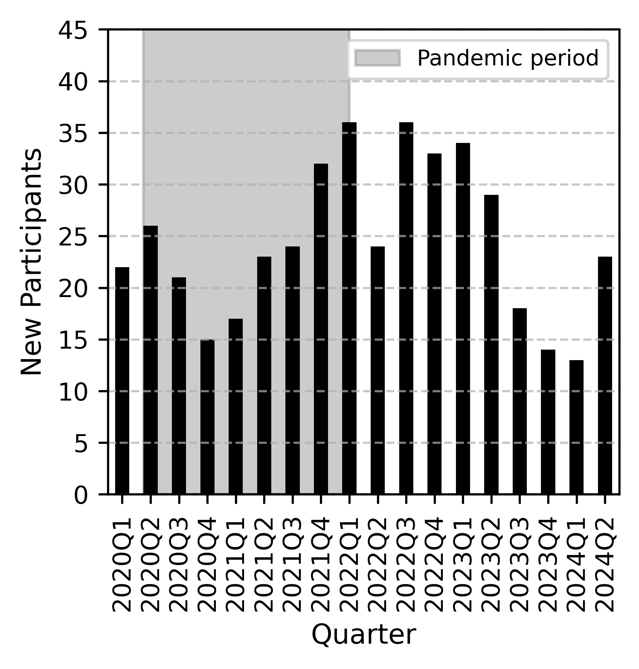


**Suppl. Fig. 5** Quarterly STI testing rates among daily PrEP users (n=453)

Bars represent the number of STI tests conducted per daily PrEP participant, per quarter. Testing rates are calculated as total tests divided by the number of daily PrEP users in each quarter. Only routine PrEP visits, unscheduled tests were not included


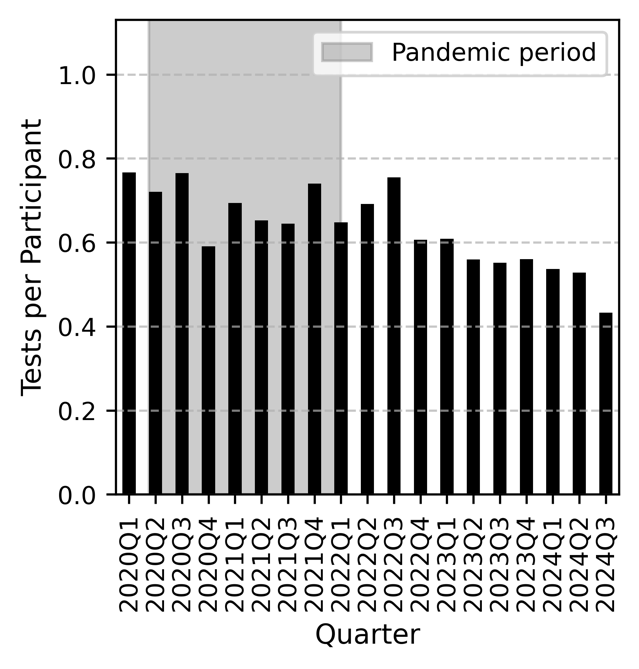


**Suppl. Fig. 6** Quarterly STI positivity rates by pathogen

The line plots show the percentage of tests returning positive results for *Chlamydia trachomatis*, *Neisseria gonorrhoeae*, Syphilis, and any STI across calendar quarters. Rates are calculated as the number of positive results divided by the total number of tests performed for PrEP User (n= 453) each quarter.


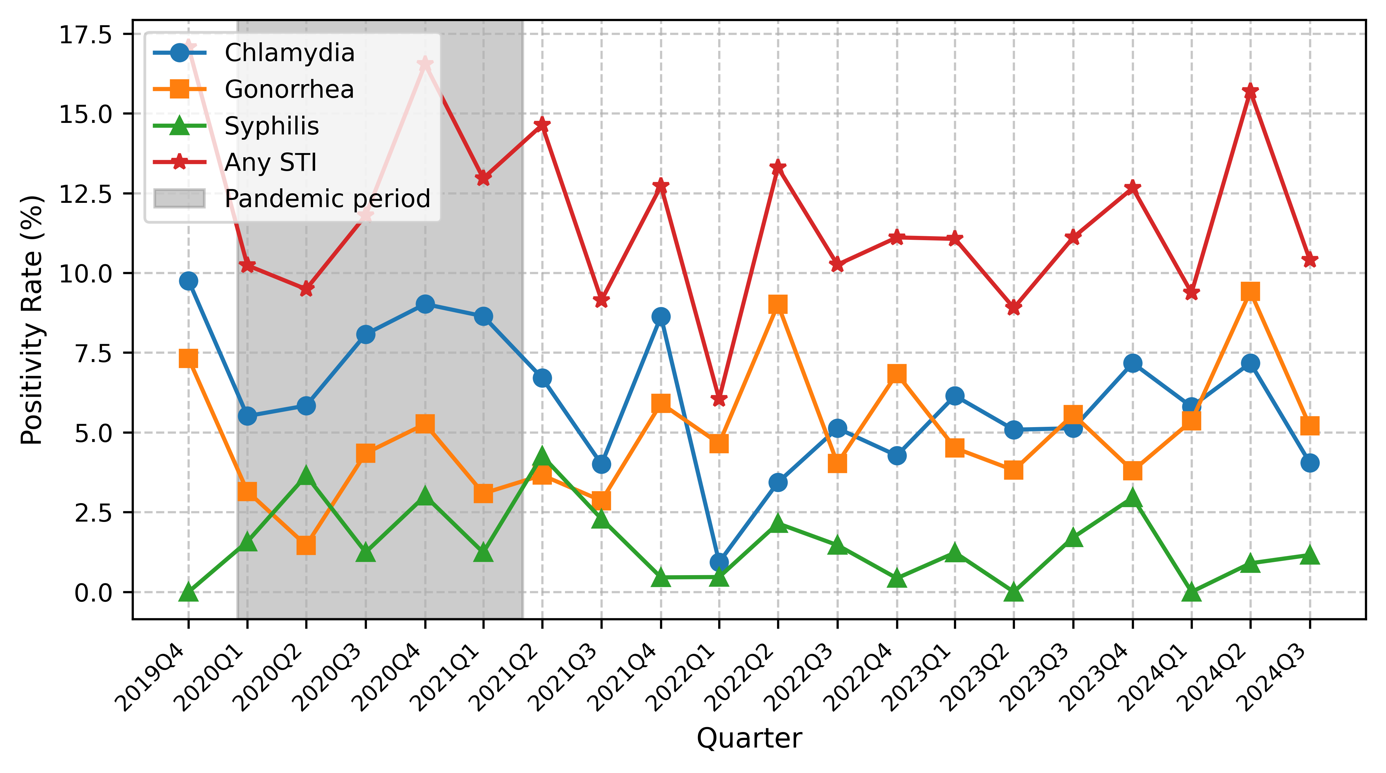


| Suppl. Table 1 Case of HIV infection | |
| --- | --- |
| Gender | male |
| Sexual orientation | MSM |
| Number of PrEP Visits | 10 |
| Number of PrEP Prescriptions | 6 |
| Number of STIs (not HIV) | 4 (2*) |
| Substance use | unknown |
| Days to diagnosis after last Prescription | 738 |
| Days on PrEP | 410 |
| Viral load by diagnosis | 311 000 c/ml |
| CD4+ cell count by diagnosis | 307 cells/µl (17%) |
| Relevant drug resistance mutations | none |
| HIV subtype | A6 |
| Initiated antiretroviral therapy | bictegravir, emtricitabine and tenofovir alafenamide |

* during analysis period

This table shows the clinical and behavioral characteristics of the participant who acquired HIV after PrEP initiation and after the analysis period

**Suppl. Fig. 7** PrEP use timeline in participant with post-PrEP HIV seroconversion

The dashed red line marks the analysis reference date. HIV diagnosis occurred 738 days after the last prescription, during a period without documented PrEP use
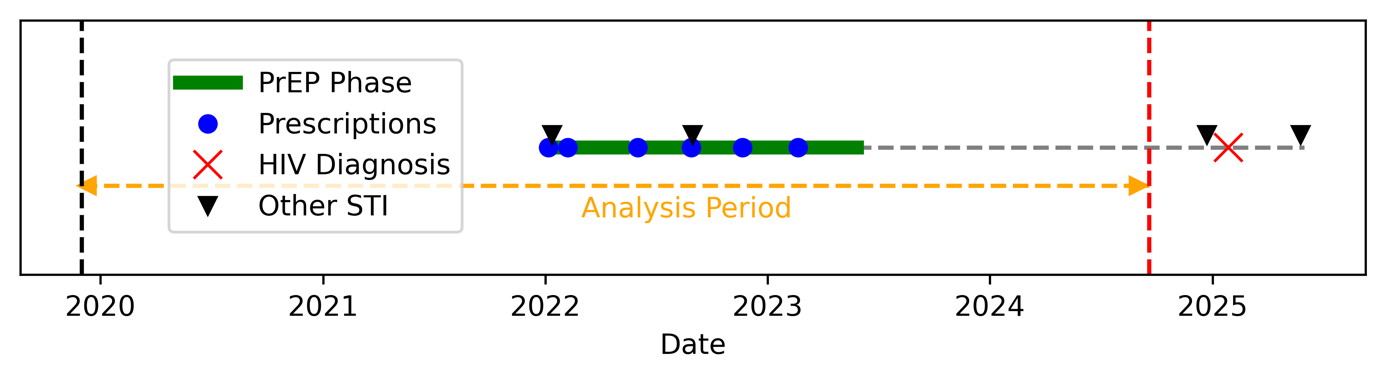


**Suppl. Fig. 8** Visualization of daily PrEP users

Each horizontal line represents a participant's documented PrEP use during the analysis period. This visualization illustrates the growth of the cohort over time and variation in individual adherence patterns among daily PrEP users (n=421)**
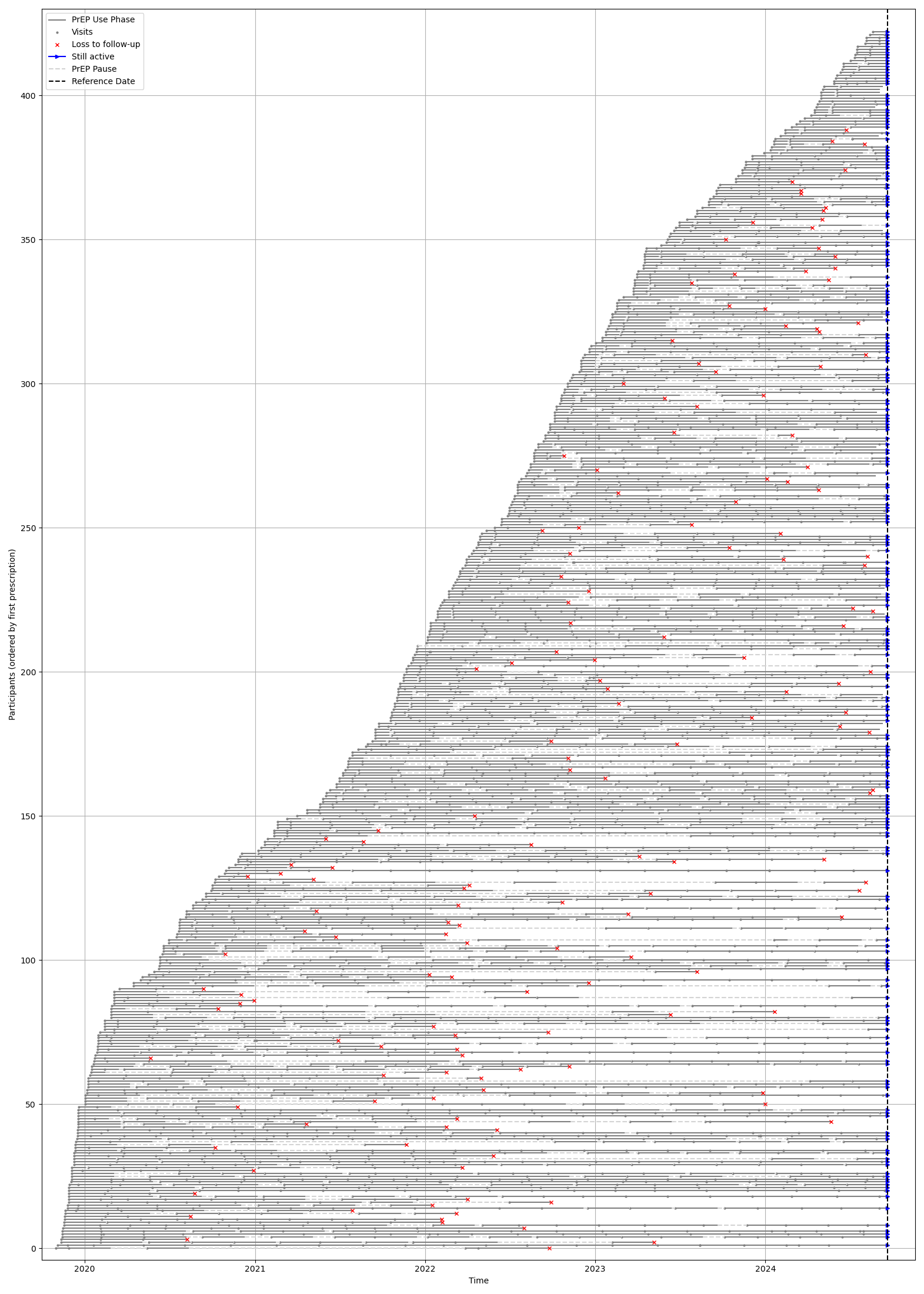
**

**Suppl. Fig. 9** Visualization of on-demand PrEP users

Each horizontal line represents a participant's documented PrEP use during the analysis period. This visualization illustrates the PrEP use patterns among on-demand PrEP users (n = 80)

**
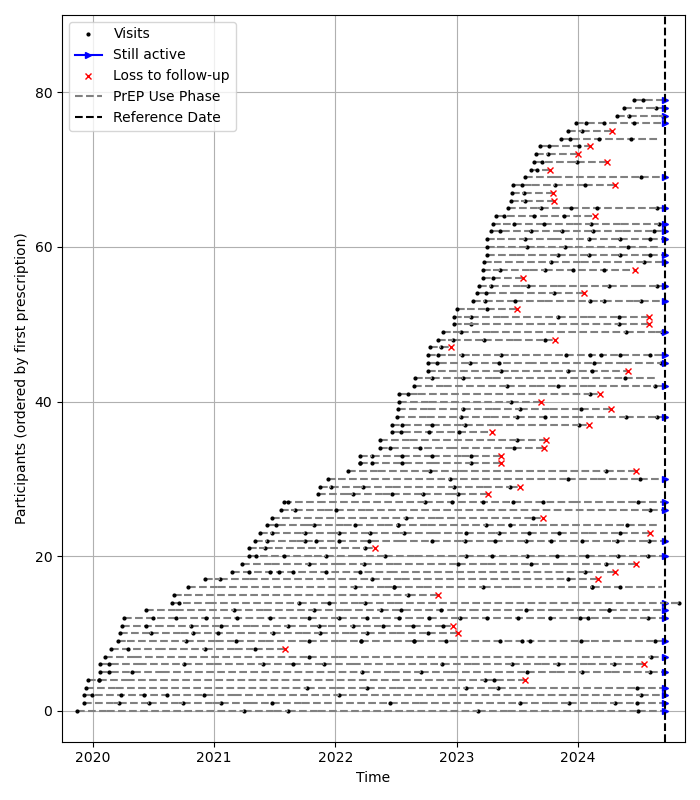
**

**Suppl. Fig. 10** Visualization of switching PrEP users

Each horizontal line represents a participant's documented PrEP use during the analysis period. This visualization illustrates the PrEP use patterns among PrEP users alternating between daily and on-demand PrEP modalities (n = 29)

**
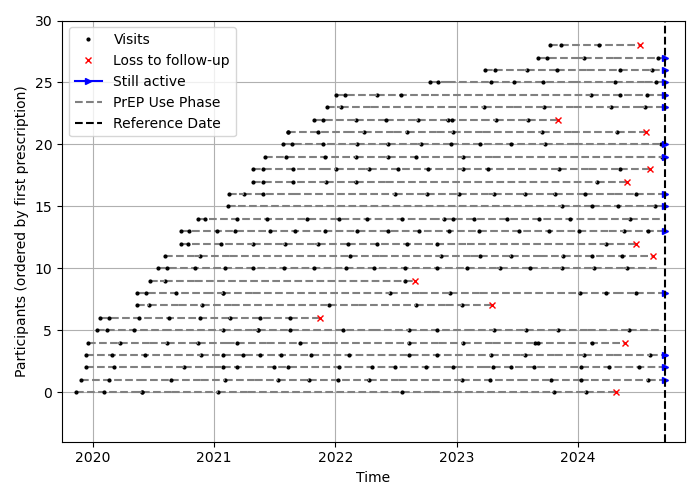
**
